# The Risk of Arrhythmias in Patients with COVID-19

**DOI:** 10.3390/biomedicines13061368

**Published:** 2025-06-03

**Authors:** Lina Haj Ali, Loredana Suhov, Adrian Apostol, Larissa Dăniluc, Oana Sandu, Carina Bogdan, Viviana Mihaela Ivan

**Affiliations:** 1 Doctoral School, “Victor Babeș” University of Medicine and Pharmacy, Eftimie Murgu Sq. No. 2, 300041 Timisoara, Romania; lina.haj-ali@umft.ro (L.H.A.); larissa.daniluc@umft.ro (L.D.); oana.ciolpan@umft.ro (O.S.); carina.bogdan@umft.ro (C.B.); 2 Department of Cardiology, Pius Brinzeu Clinical Emergency County Hospital Timisoara, 300736 Timisoara, Romania; adrian.apostol@umft.ro (A.A.); ivan.viviana@umft.ro (V.M.I.); 3 Department VII, Internal Medicine II, Discipline of Cardiology, “Victor Babeș” University of Medicine and Pharmacy, Eftimie Murgu Sq. No. 2, 300041 Timisoara, Romania

**Keywords:** COVID-19, inflammatory markers, arrhythmia, atrial fibrillation

## Abstract

**Background and Objectives:** Cardiac arrhythmias during the SARS-CoV-2 infection may occur due to the direct impact of the virus on the respiratory and cardiovascular systems, as well as through the broader effects of systemic inflammation, or a combination of both. Additional mechanisms include the proarrhythmic effects of COVID-19 pharmacotherapies, drug–drug interactions, and associated autonomic dysfunction. To improve future risk stratification and clinical management, it is essential to accurately assess the risk of arrhythmia in the context of severe infections, in order to develop diagnostic and therapeutic algorithms that support the fastest and safest possible interventions. **Materials and Methods:** This retrospective observational study included 151 patients diagnosed with COVID-19, who were hospitalized at the Cardiology Clinic of the Timisoara County Emergency Hospital between 2020 and 2022. **Results:** The most common arrhythmia observed was atrial fibrillation. Elevated inflammatory markers were associated with a higher risk of arrhythmias and increased mortality. **Conclusions:** The onset of sepsis, as indicated by the laboratory markers, was associated with increased incidence of arrhythmias and unfavorable outcome of the disease.

## 1. Introduction

COVID-19 was first identified in 2019 as a disease caused by Coronavirus-2 primarily affecting the respiratory system [1]. Global data indicate that the majority of patients experience mild to moderate forms of the disease while a small percentage develop severe illness [1,2]. The most feared complications include multiple organ failure in 5% of severe cases, sepsis, coagulopathies, pulmonary and cardiovascular complications, which are in direct association with the inflammatory syndrome associated with the disease [1].

Reduced expression of angiotensin-converting enzyme 2 (ACE2), expressed on cell membranes in the heart and lungs, has been observed in certain pathological conditions and viral infections in laboratory animal models [3,4]. ACE 2 has protective effects against cellular injury, which may partially explain the more severe lesions in patients with pre-existing cardiovascular disease with drug-modified expression of this enzyme [4].

ACE2, mainly expressed in pulmonary alveoli type 2, myocardial cells and kidney proximal tubules, serves as an entry receptor for the SARS-CoV-2 Spike (S) protein. As a consequence, an imbalance in the renin-angiotensinogen system occurs, triggering a severe inflammatory response in the lungs mediated by angiotensin 2, which in turn activates the production of other markers such as IL 8 and Monocyte chemoattractant protein-1 (MCP1) that are expressed in endothelial cells, and decrease the synthesis of angiotensin 1–7 with an anti-inflammatory, antifibrotic role with consequent exacerbation of the inflammatory response [1].

The onset of hypoxemia in the evolution of the disease may favor the production of adenosine triphosphate (ATP) through the glycolysis pathway, primarily mediated by pyruvate kinase, PKM2 promotes glycolysis and increases lactate levels, additionally it mediates the synthesis of hypoxia-inducible factor-1a (HIF1a) which in turn leads to the secretion of other pro-inflammatory cytokines such as IL1β, IL6, and secondary production of IL 10. These cytokines may be the main contributors to acute respiratory distress syndrome and inflammatory pulmonary infiltrates [5].

Type 1 and 2 pneumocytes, along with alveolar macrophages, are mainly affected. Increased production of interferon I, and III has been observed, but with a more attenuated response that would easily multiply the virus in lung tissue. The mentioned interleukins and tumor necrosis factor (TNF), Interferon I and III (INF I and III), Monocyte chemoattractant protein-1 (MCP1), C-X-C motif chemokine ligand 1 (CXCL1), C-X-C motif chemokine ligand 5 (CXCL5) are the main markers involved in regulating the innate immune response to the SARS-CoV-2 infection [6].

In individuals without comorbidities, an effective inflammatory response can lead to the recruitment of virus-specific T lymphocytes to the site of infection. This localized immune activation plays a crucial role in viral clearance and may help prevent the progression of the disease and the occurrence of complications [7,8].

Some studies confirm that in patients with severe forms of the disease there is a specific subpopulation of macrophages; these patients exhibit a significant increase in CD14+CD16+ monocytes that secrete cytokines—this monocyte subset is characteristic for complicated forms of the COVID-19 infection [9]. B lymphocyte-mediated humoral immunity functions through the production of neutralizing antibodies. In severe forms of the disease, B cells undergo differentiation into plasma cells. Additionally, the number of natural killer (NK) cells is reduced, while neutrophil counts are elevated [10].

Envelope protein E is a membrane-associated protein that is highly expressed in host cells during viral replication. A small fraction is incorporated into the virion envelope, while the majority localizes to the Golgi apparatus or the endoplasmic reticulum (ER)—Golgi intermediate compartment of the host cell. Protein E contributes to the pathogenesis of several coronaviruses, and studies have shown that it can induce alterations in ionic permeability [11].

In the context of COVID-19, inflammatory cytokines, particularly TNF-α, IL-1, and IL-6, contribute to the development of arrhythmias through multiple mechanisms [12]. These cytokines affect the electrophysiological properties of myocardial cells by altering the ion channel function and disrupting the normal balance of ion flux, leading to a prolongation of action potentials and irregular myocardial repolarization. This creates a proarrhythmic environment, particularly in patients with pre-existing cardiovascular conditions or those experiencing a severe viral infection. In addition, cytokine-induced inflammation may exacerbate dysfunction of the autonomic nervous system, further promoting both atrial and ventricular arrhythmias. These findings underscore the link between systemic inflammation and electrophysiological disturbances, highlighting the importance of closely monitoring inflammatory markers in patients at increased risk for arrhythmic events [12].

We aim to study the arrhythmic risk in this disease by considering the cardiovascular complications from a COVID-19 infection: arrhythmias triggered by myocardial injury or secondary to inflammatory syndrome. Myocarditis, acute coronary syndrome, acute or exacerbated heart failure, and acute circulatory collapse are the main causes of arrhythmias [13,14].

Atrial fibrillation (AF) is one of the most frequently encountered arrhythmias in patients with cardiovascular complications, including those with COVID-19. Several clinical parameters are known to influence the likelihood of spontaneous conversion to sinus rhythm and are relevant for risk stratification in this population. These include a reduced left ventricular ejection fraction, which is associated with lower conversion rates, and increased left atrial size, particularly a diameter greater than 41 ± 7 mm. The shorter the duration of an AF episode (especially under 24 h), the higher the probability of a successful conversion. Additional predictive factors include a lower atrial fibrillatory rate (AFR), elevated plasma levels of atrial natriuretic peptide (ANP) above 300 ng/L, which reflect atrial structural activity, and episodes occurring during sleep. These markers, which indicate the degree of atrial remodeling and myocardial stress, may provide valuable information when evaluating arrhythmic risk in patients with COVID-19 and systemic inflammation [15].

Persistent, multi-organ dysfunction following COVID-19 infection is referred to as Long COVID. The mechanisms underlying myocyte injury in this condition are not yet fully understood. However, the cytokine storm and endothelial dysfunction that occur during disease progression may contribute to a myocardial lesion [16]. These complications can also predispose patients to the development of arrhythmias.

The proarrhythmic environment associated with COVID-19 infection—characterized by hypoxia, fever, electrolyte imbalance (particularly hyper/hypokalemia), as well as the use of antiviral or antibiotic agents, and various supportive therapies, can also favor the occurrence of ventricular arrhythmias. This risk increases in patients with comorbidities or genetic syndromes such as QTL or Brugada Syndrome [17]. Studies indicate that reduced heart rate variability (HRV) is strongly associated with autonomic nervous system dysfunction, a condition that may increase the risk of ventricular arrhythmias [18]. Additionally, it is important to note that poorly controlled hypertension significantly elevates the risk of cardiovascular mortality and cerebrovascular events (stroke) [19].

Patients with comorbidities, such as obesity, chronic lung disease, diabetes and kidney diseases, are prone to more rapid disease progression and a poorer prognosis [20]. They are also more likely to experience electrolyte imbalances, which can consequently lead to ventricular arrhythmias. Dyselectrolytemias in COVID-19 have various causes, with hypokalemia being among the most frequently observed. Additionally, the dysregulation of the renin–angiotensin–aldosterone system (RAAS) during the course of the disease may have significant clinical implications [21].

Disturbances in phosphocalcic metabolism can precipitate endo-myocardial calcifications, which further impair tissue depolarization and conduction pathways, thereby increasing the risk of arrhythmias [22,23].

The objective of this study is to identify patients with COVID-19 who are at increased risk of developing arrhythmias, with particular attention to those exhibiting inflammatory syndrome. The analysis focuses on comorbidities and complications arising during the course of the disease, in order to support the timely and targeted implementation of prophylactic measures.

## 2. Materials and Methods

### 2.1. Study Population

The study was conducted at the Timisoara County Emergency Hospital, and included 151 patients who were hospitalized in the Cardiology Clinic between 2020 and 2022. Participants were classified into two groups based on disease severity and the presence of sepsis. Group 1 included individuals with severe or critical forms of the disease, who also met the diagnostic criteria for sepsis. Group 2 consisted of patients with mild to moderate COVID-19, without any clinical or laboratory evidence of sepsis. The patients were categorized according to the severity of COVID-19 at admission, as follows:

- Mild: Patients with minimal symptoms (mild fever, cough, myalgias).
- Moderate: Patients with clinical and imaging signs (chest X-ray or chest CT scan) suggestive of pneumonia.
- Severe: Patients with acute respiratory failure—respiratory rate > 30 breaths/min, oxygen saturation < 93% in ambient air.
- Critical: Patients who required mechanical ventilation or presented with multiple organ failures and were admitted to the intensive care unit (ICU).

Sepsis was diagnosed based on the Sepsis-3 definition, which describes it as a potentially fatal condition characterized by organ dysfunction resulting from an abnormal immune response to infection. The presence and severity of organ dysfunction were assessed using the SOFA (Sequential Organ Failure Assessment) score, according to the criteria established by the Third International Consensus on Sepsis and Septic Shock [24].

### 2.2. Inclusion and Exclusion Criteria

Inclusion criteria: Patients with a positive polymerase chain reaction (PCR) test for COVID-19 infection, hospitalized in the cardiology department, with cardiovascular risk factors presented such as: age > 55years in men and >65years in women, arterial hypertension, smoking, dyslipidemia, diabetes mellitus, obesity, and alcohol consumption. We included patients with coronary artery disease, heart failure with reduced ejection fraction (EF ≤ 40%), chronic kidney disease, or history of stroke.

Exclusion criteria: Patients with thyroid disorder, due to the potential risk of thyrotoxicosis or treatment related overdose, which could precipitate arrhythmias. Patients with a history of antiarrhythmic therapy were also excluded, as these medications may either suppress arrhythmias related to the underlying disease or, through adverse effects, independently induce arrhythmias. In addition, patients who did not provide informed consent were excluded from the study.

### 2.3. Patient Investigation

Biological samples: Diagnostic testing included a PCR test for SARS-CoV-2 infection and a panel of inflammatory markers, including erythrocyte sedimentation rate (ESR), C-reactive protein, IL 6, and fibrinogen. D-dimer levels were measured upon hospital admission, along with cardiac and liver enzymes, serum creatinine, electrolyte panel, and coagulation profile, to assess for the potential occurrence of disseminated intravascular coagulation (DIC)—an important negative prognostic factor in the clinical course of COVID-19. The value of procalcitonin was also monitored, as a biomarker associated with severe bacterial coinfection and systemic inflammation.

Paraclinical investigations: Arrhythmias were initially diagnosed at admission using a standard 12-lead electrocardiogram (ECG), systematically performed as part of the comprehensive clinical evaluation for all patients. This was complemented by the use of a 24 h, 3-channel Holter ECG monitoring as an additional method for extended rhythm assessment. Continuous telemetry monitoring was implemented in all cases where closer cardiac surveillance was clinically indicated, such as in patients admitted to intensive care units or those presenting with signs of hemodynamic instability. The use, timing, and frequency of these diagnostic modalities were determined based on the clinical presentation and the patient’s condition, aiming to ensure accurate diagnosis and appropriate therapeutic management. Additional investigations, including transthoracic echocardiography, chest radiography, and thoracic computed tomography, were also performed.

Vital parameters such as heart rate, blood pressure, oxygen saturation, body temperature, hourly and 24 h diuresis were monitored. Treatment was adjusted according to the patient’s clinical evolution.

### 2.4. The Arrhythmias Studied

Cardiac arrhythmias were documented as follows:

- Supraventricular arrhythmias, including atrial fibrillation, atrial flutter, and other forms of supraventricular tachycardia.
- Ventricular arrhythmias, including ventricular extrasystoles, ventricular tachycardia, ventricular fibrillation, and torsades de pointes.
- Bradyarrhythmias, including sinus bradycardia and atrioventricular (AV) blocks (first-degree, second-degree, and complete AV block).

### 2.5. Statistical Analysis

Statistical analysis was performed using GraphPad Prism version 10.4.1 and Microsoft Excel (2016). The normality of data distribution was assessed using the Shapiro–Wilk test. Continuous variables with a normal (Gaussian) distribution were reported as means and standard deviations (SDs), while non-normally distributed variables were presented as medians and interquartile ranges (IQRs). Descriptive statistics were used to summarize demographic and clinical characteristics. Continuous variables (e.g., age, biological parameter values) were expressed as means ± SD, and categorical variables (e.g., gender, comorbidities, complications, and mortality) as frequencies and percentages. For group comparisons, continuous variables were analyzed using linear model ANOVA, while categorical variables were compared using Pearson’s chi-squared test or Fisher’s exact test, as appropriate. For the comparisons of the two groups, independent t-tests samples were used. A multivariable logistic regression analysis was performed to identify independent predictors of mortality, with mortality coded as “Deceased = 1.” Variables with a *p*-value < 0.1 in the univariate analysis were included in the multivariable model. Results were reported as odds ratios (ORs) with 95% confidence intervals (CIs). A *p*-value < 0.05 was considered statistically significant. Forest plots were generated using version 3.10 of Python, with the Matplotlib and Seaborn libraries, based on the statistical results from previous analyses.

### 2.6. Ethical Consideration

Informed consent was obtained on admission to the cardiology clinic. Patients expressed consent for future research for scientific purposes in accordance with ethical guidelines. The study was approved by the hospital ethics committee with approval number 12/27.01.2020. All clinical and paraclinical investigations were performed by experienced specialists.

## 3. Results

### 3.1. Study Group Description

A total of 151 patients diagnosed with COVID-19 were evaluated. Two groups were established: group 1 included 92 patients who presented clinical and paraclinical criteria for sepsis, while group 2 included 59 patients without signs of sepsis. The mean age of the participants in group 1 was 70.9 ± 11.3 while in group 2 was 73.9 ± 9.5. The majority of patients in group 1 were male, while in group 2 they were female. The effect of comorbidities on disease progression and possible complications was also assessed. 68.5% of patients in group 1 had acute kidney injury (AKI) compared to 13.6% in group 2 (*p* = 0.00). Table 1 details the demographic data, presence of comorbidities and some of the complications.

### 3.2. Arrhythmias in COVID-19 Infection

**Figure 1:**
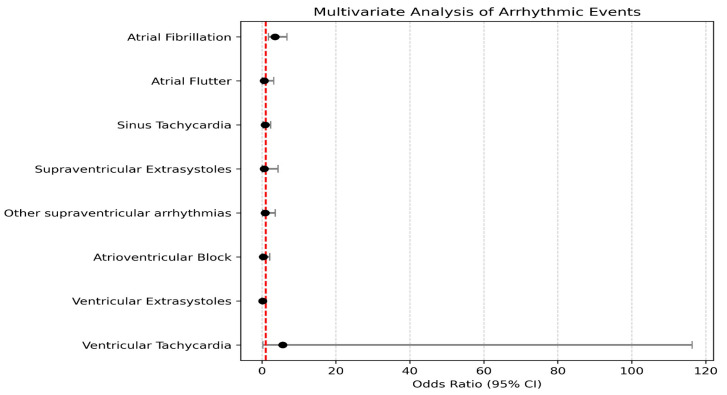
Multivariate analysis of arrhythmic events in the studied groups.

In the multivariate analysis of arrhythmic events, atrial fibrillation showed a significant association with disease severity, with an OR of 3.50 (95% CI: 1.80–6.80, *p*-value = 0.001), indicating a risk approximately 3.5 times higher for patients in group 1. In contrast, other arrhythmic events, including atrioventricular block (OR = 0.32, *p*-value = 0.322), ventricular tachycardia (OR = 5.62, *p*-value = 0.251), and ventricular extrasystoles (OR = 0.23, *p*-value = 0.068), did not demonstrate statistically significant associations with COVID-19 severity. These results suggest that COVID-19 severity is significantly associated only with the risk of atrial fibrillation among the arrhythmic events analyzed. These results are found in Table 2 and Figure 1.

### 3.3. Inflammatory Syndrome and Mortality in COVID-19

We analyzed the impact of inflammatory markers such as CRP, IL-6, on disease progression. We observed increased values of inflammatory markers in patients with sepsis: the mean value of C-reactive protein was 195 ± 121.90 in group 1 and 98.32 ± 97.96 in group 2—a statistically significant difference (*p* < 0.0001)—the same results for interleukin 6 (*p* < 0.0001). The analyzed data are presented in Table 3.

We investigated the effect of disease progression and the occurrence of sepsis on increasing mortality risk. According to our analyzed data the number of deaths was higher in group 1 compared to group 2, the data were statistically significant (*p* < 0.0001). In the context of this study, mortality refers specifically to in-hospital mortality, as seen in Table 4.

**Figure 2:**
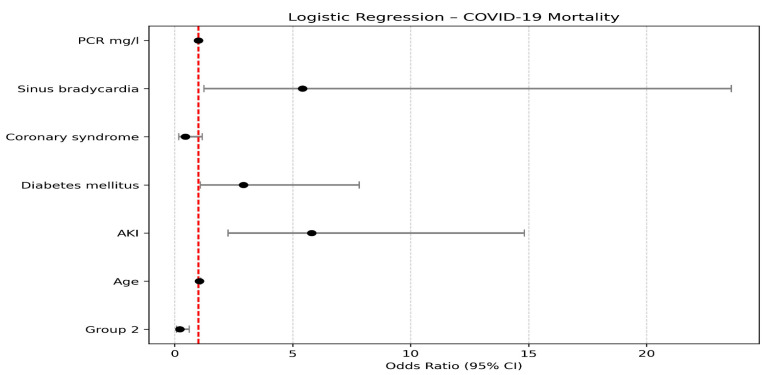
Logistic Regression analysis of mortality in COVID-19 patients.

The logistic regression analysis identified several statistically significant predictors of mortality. Sinus bradycardia was significantly associated with an increase in mortality, with its presence increasing the log odds by 1.689 (Odds Ratio = 5.414, *p* = 0.025; 95% CI: 0.217 to 3.161), suggesting that patients with sinus bradycardia have approximately 5.4 times higher odds of death compared to those without this condition. Diabetes mellitus was also significantly associated with increased mortality (Estimate = 1.069, Odds Ratio = 2.914, *p* = 0.034; 95% CI: 0.082 to 2.057), indicating nearly a three-fold increase in the odds of death among patients with diabetes. The presence of acute kidney injury (AKI) emerged as one of the strongest predictors; patients with AKI experienced a 1.758 unit increase in log odds (Odds Ratio = 5.798, *p* < 0.001; 95% CI: 0.819 to 2.696), reflecting nearly sixfold higher odds of death. Age was a significant predictor as well (Estimate = 0.049, Odds Ratio = 1.050, *p* = 0.022; 95% CI: 0.007 to 0.091), implying that each additional year of age increases the odds of death by approximately 5%. Finally, membership in Group (2) was associated with a significant reduction in mortality risk (Estimate = −1.506, Odds Ratio = 0.222, *p* = 0.004; 95% CI: −2.535 to −0.477), suggesting that patients in Group (2) had about 78% lower odds of death relative to the reference group. These results are illustrated in Table 5. and Figure 2.

We analyzed the correlation between increased C-reactive protein and mortality: 71.88% of the deaths in group 1 and 36.36% in group 2 had C-reactive protein values above 100 mg/L (*p* = 0.03). Also, 78.13% of the deaths in group 1 had acute kidney injury, by comparison with 36.36% in group 2, the data were statistically significant and are shown in Table 6.

## 4. Discussion

In this study, 53.26% of the patients with severe forms of the disease complicated with sepsis were male, whereas the majority of those without superinfections or hemodynamic deterioration were female. This has also been observed by Jin et al., where the majority of cases, with severe forms of the disease and deaths, have been recorded in males [25].

In our study most of the patients presented with comorbidities, where chronic coronary syndrome was observed in approximately half of the patients in both groups. Elderly, male, and obese patients tend to develop complications more frequently, including multiple organ failure, as observed by Hu et al. [26].

Diabetes, chronic cardiovascular disease, and obesity were frequently associated with severe disease forms, which can be attributed to the chronic inflammation already present in this category of patients, as mentioned by Russell et al. [16]. Chronic obstructive lung disease often progresses with respiratory distress and is linked to poorer outcomes. Increased mortality has been documented by Sanyaolu et al. in patients with chronic kidney disease and malignancies. These patients, additionally, face a higher risk of contracting the infection [20].

We analyzed the types of arrhythmias that occurred and atrial fibrillation was the most predominant arrhythmia in both study groups. Wang et al. highlight that long-term exposure to elevated IL-6 can alter the intracellular calcium concentration, potentially leading to various arrhythmias. Similar effects have been described for IL1β and TNF-α [27].

Lavelle et al. demonstrated that atrial arrhythmias are the most frequently documented arrhythmias in patients with severe forms of the disease. Atrial fibrillation and atrial flutter were more common in older patients and those with elevated inflammatory markers. These arrhythmias were also associated with increased mortality [28].

Musikantow et al. reported that 61% of patients who developed atrial fibrillation or atrial flutter during hospitalization had a prior history of atrial arrhythmias [29].

In our study 13.04% of patients with sepsis exhibited sinus tachycardia. Pandat et al. maintain that sinus tachycardia has been the most common arrhythmia, which is in most cases secondary to hypovolemia, hypoperfusion, fever, dehydration. Atrioventricular nodal reentrant tachycardia was observed more frequently in younger patients [30].

Only 3.26% of our patients experienced ventricular tachycardia, and 2.17% had ventricular extrasystoles. Babapoor-Farrokhran et al. confirm the low incidence of these arrhythmias, which tend to occur in patients with heart disease, most commonly heart failure, left ventricular systolic dysfunction or myocardial infarction, and with risk factors such as diabetes or obesity. The proarrhythmic effects of certain antibiotics and antivirals, mediated through QT interval prolongation, has also been documented [14].

On the other hand, 16.30% of patients with severe disease presented with sinus bradycardia. An overview by Douedi et al. shows that 8% of hospitalized patients with severe forms had severe sinus bradycardia, and 8% experienced first or second-degree atrioventricular block during hospitalization; these patients were treated with antivirals. The mechanisms by which bradyarrhythmias occur are not yet completely known but a strong association between Interleukin-6 and bradycardia has been documented. The severe inflammatory response may directly affect the sinoatrial node, enhancing vagal tone and, thus, promoting bradycardia. Additionally, hypoxia, metabolic disturbances, and electrolyte imbalances can add to the potential causes [31]. In a retrospective study by Alsowaida et al., which included 1635 hospitalized COVID-19 patients, the incidence of bradycardia was 37.1%, with 9.7% exhibiting moderate bradycardia and 0.7% classified as severe. Patients with risk factors such as age > 65 years, hypertension, and obesity were more likely to develop bradycardia following the administration of remdesivir [32].

In our study, 68.48% of patients with sepsis developed acute kidney injury (AKI), compared to 13.56% of patients without clinical signs of sepsis. Legrand et al. report that 30–50% of patients with sepsis develop varying degrees of AKI, with early signs of renal impairment, such as hematuria and proteinuria, observed in 40% and 26% of cases, respectively [33]. A clear association was identified between the severity of respiratory failure and the occurrence of AKI. The severe form of AKI was most frequently observed in conjunction with worsening respiratory function and the need for mechanical ventilation. This correlation was documented by Hirsch et al., who found that AKI occurred in 65.5% of ventilated patients, compared to only 6.7% of non-ventilated patients [34].

In our study 78.13% of deaths in patients with sepsis had AKI. Kolhe et al. confirm that onset of AKI is a risk factor for mortality [35]. In our study, patients with severe forms of the disease had significantly elevated CRP levels, with 71.88% of deceased patients with sepsis exhibiting CRP values greater than 100 mg/L. L. Wang confirmed the association between elevated CRP levels and the severity of pulmonary lesions [36]. Similarly, increased levels of interleukin-6 (IL-6) are strongly correlated with disease progression to severe forms and the development of respiratory distress, as reported by Ulhaq et al. [37]. Mortality in patients with COVID-19 and sepsis was 59%. Age, maximum procalcitonin value above 2 ng/mL, deterioration of renal function, necessity of invasive ventilation, and septic shock were identified as risk factors for mortality in hospitalized patients in the study by Heubner et al. [38]. Arrhythmias were significantly more prevalent in patients with worsening clinical progression and poor prognosis (48%) compared to those with a favorable outcome (6%). These arrhythmias may exacerbate metabolic disturbances, hypoxia, and neurohormonal imbalances, thereby contributing to an increased risk of mortality in patients with severe forms of the disease [39]. The mortality rate in patients with sepsis in our study was 69.57%.

Based on the findings of this study, we recommend that cardiologists closely monitor patients with pre-existing cardiovascular comorbidities, diabetes, and obesity, as these individuals are at increased risk for arrhythmias and other complications. In patients with sepsis or elevated inflammatory markers, continuous cardiovascular monitoring is essential, with a particular focus on the early detection of arrhythmias. Given the potential proarrhythmic effects of certain COVID-19 treatments, careful management is necessary, especially in those with underlying cardiovascular disease. Furthermore, controlling inflammation and effectively managing respiratory and renal insufficiency may help reduce arrhythmic risk and improve prognosis. Early detection and prompt intervention are crucial for improving survival outcomes in this high-risk patient population.

### Limitations of the Study

This retrospective study has several limitations. First, the difficulty of Holter monitoring in these patients made it challenging to accurately assess the duration and burden of these arrhythmias. Second, the relatively small sample size may have limited the generalizability of our results; therefore, future studies with larger patient cohorts are needed to better investigate the effect of inflammatory syndrome and COVID-19-specific immunologic reactions on the increased arrhythmic risk. Additionally, the inclusion of only patients with cardiovascular risk factors admitted to the cardiology department may have introduced selection bias, potentially limiting the applicability of our findings to the general COVID-19 population. Furthermore, the assessment of inflammation in this study primarily relied on inflammatory markers such as C-reactive protein (CRP) and IL-6, which may not fully capture the complexity of the inflammatory process. Even after adjusting for several potential confounding factors, residual confounding may still exist. Unmeasured or inadequately controlled variables, such as genetic predispositions, lifestyle factors, and pre-existing inflammatory conditions, could influence both inflammation and the risk of arrhythmias, potentially confounding the observed relationship.

## 5. Conclusions

Our study highlights a correlation between the severity of the inflammatory response in COVID-19 and the occurrence of arrhythmias. This association appears more pronounced in patients with pre-existing comorbidities, particularly cardiovascular disease and diabetes mellitus, as well as in severe forms of the disease complicated by sepsis. The presence of acute kidney injury and hydroelectrolyte imbalances was also associated with poorer clinical outcomes. Atrial fibrillation emerged as the most frequently observed arrhythmia across both study groups. These findings underscore the potential role of systemic inflammation and multi-organ involvement in the development of arrhythmic events during COVID-19, although causality cannot be established due to the observational design of the study. The results support the need for close cardiac monitoring and early intervention, particularly in high-risk patients. Given the high incidence of arrhythmias especially atrial fibrillation, observed in this study, further research is warranted to develop evidence-based management strategies that incorporate inflammatory status, electrolyte disturbances, and renal dysfunction, especially in patients with multiple comorbidities.

## Figures and Tables

**Table 1:** Characteristics of the study population.

Demographic Data, Comorbidities, and AKI.	1 (N = 92)	2 (N = 59)	Total (N = 151)	*p* Value
**Age**				0.086 ^1^
Mean (SD)	70.9 (11.3)	73.9 (9.5)	72.1 (10.7)	
Range	36.0–94.0	50.0–93.0	36.0–94.0	
**Gender**				0.486 ^2^
F	43.0 (46.7%)	31.0 (52.5%)	74.0 (49.0%)	
M	49.0 (53.3%)	28.0 (47.5%)	77.0 (51.0%)	
**Chronic coronary syndrome**				0.611 ^2^
No	46.0 (50.0%)	27.0 (45.8%)	73.0 (48.3%)	
Yes	46.0 (50.0%)	32.0 (54.2%)	78.0 (51.7%)	
**Diabetes mellitus**				0.889 ^2^
No	68.0 (73.9%)	43.0 (72.9%)	111.0 (73.5%)	
Yes	24.0 (26.1%)	16.0 (27.1%)	40.0 (26.5%)	
**Chronic kidney disease**				0.057 ^2^
No	60.0 (65.2%)	47.0 (79.7%)	107.0 (70.9%)	
Yes	32.0 (34.8%)	12.0 (20.3%)	44.0 (29.1%)	
**Stroke**				0.837 ^2^
No	72.0 (78.3%)	47.0 (79.7%)	119.0 (78.8%)	
Yes	20.0 (21.7%)	12.0 (20.3%)	32.0 (21.2%)	
**AKI**				<0.001 ^2^
No	29.0 (31.5%)	51.0 (86.4%)	80.0 (53.0%)	
Yes	63.0 (68.5%)	8.0 (13.6%)	71.0 (47.0%)	

^1^ Linear Model ANOVA ^2^ Pearson’s Chi-squared test SD: standard deviation. AKI: Acute kidney Injury.

**Table 2:** Multivariate analysis of arrhythmic events in the studied groups.

	Odds Ratio	95% CI	*p*-Value
Atrial Fibrillation	3.50	1.80–6.80	0.001
Atrial Flutter	0.63	0.12–3.17	0.577
Sinus Tachycardia	0.89	0.34–2.32	0.815
Supraventricular Extrasystoles	0.64	0.09–4.37	0.654
Other supraventricular arrhythmias	0.88	0.21–3.58	0.856
Atrioventricular Block	0.32	0.05–2.10	0.322
Ventricular Extrasystoles	0.23	0.05–1.10	0.068
Ventricular Tachycardia	5.62	0.27–116.3	0.251

**Table 3:** Analysis of C-reactive protein and Interleukin-6 values in both groups.

Inflammatory Markers	Group 1	Group 2	*p* Value
C-reactive protein (mg/L)	195 ± 121.90	98.32 ± 97.96	<0.0001 ****
Interleukin-6 (pg/mL)	108.1 ± 75.50	83.32 ± 33.60	<0.0001 ****

Data are presented as mean value ± standard deviation; **** *p* value < 0.01.

**Table 4:** Mortality analysis in the studied group.

	Group 1	Group 2	*P* Value
**Mortality**	64/92 (69.57%)	11/59 (18.64%)	<0.0001 ****

Data are presented as number and percentage; **** *p* values < 0.01.

**Table 5:** Logistic Regression analysis of mortality in COVID-19 patients.

Predictor	Estimate	SE	Odds Ratio	Z	*p*-Value	95% CI (β)
**Intercept**	−4.466	1.651	0.011	−2.705	0.007	(−7.701, −1.231)
**PCR mg/L**	0.004	0.002	1.004	1.736	0.083	(−0.000, 0.008)
**Sinus bradycardia**	1.689	0.751	5.414	2.249	0.025	(0.217, 3.161)
**Coronary syndrome**	−0.786	0.479	0.456	−1.640	0.101	(−1.724, 0.153)
**Diabetes mellitus**	1.069	0.504	2.914	2.123	0.034	(0.082, 2.057)
**AKI**	1.758	0.479	5.798	3.672	<0.001	(0.819, 2.696)
**Age**	0.049	0.021	1.050	2.297	0.022	(0.007, 0.091)
**Group 2**	−1.506	0.525	0.222	−2.869	0.004	(−2.535, −0.477)

**Table 6:** Correlation between C-reactive protein value and onset of acute kidney injury and mortality.

	Group 1	Group 2	*p* Value
**CRP > 100mg/L**	71.88%	36.36%	0.03 *
**Acute kidney injury**	78.13	36.36%	0.0086 **

Data are presented as percentages; * *p* values < 0.05; ** *p* values < 0.01; CRP-C reactive protein.

## Data Availability

Data are contained within the article.

## Abbreviations

The following abbreviations are used in this manuscript:

ACE 2	Angiotensin-converting enzyme 2
IL 8	Interleukin 8
MCP1	Monocyte chemoattractant protein-1
ATP	Adenosine triphosphate
PKM2	M2 isoform of pyruvate kinase
HIF1a	Hypoxia-inducible factor-1a
IL1β	Interleukin 1β
IL6	Interleukin 6
IL10	Interleukin 10
TNF	Tumor necrosis factor
INF I, III	Interferon
CXCL1	C-X-C motif chemokine ligand 1
CXCL5	C-X-C motif chemokine ligand 5
CD14+	Cluster of differentiation 14
CD16+	Cluster of differentiation 16
CRP	C-reactive protein
ESR	Erythrocyte sedimentation rate
